# Supplementary material for: Patient Heterogeneity in Acute Myeloid Leukemia: Leukemic Cell Communication by Release of Soluble Mediators and Its Effects on Mesenchymal Stem Cells
Source: Diseases. 2021 Oct 16;9(4):74. doi: 10.3390/diseases9040074 (PMC8544451; doi:10.3390/diseases9040074)
Supplement: Supplementary file 1 [file diseases-09-00074-s001.zip › diseases-1400244-RevisedVersion-SUPPLEMENTARY-211007.pdf]

SUPPLEMENTARY INFORMATION

Effects of constitutive soluble mediator release by acute myeloid leukemia cells on the proteomic profiles of bone marrow mesenchymal stem cell; a focus on patient heterogeneity

Elise Aasebø, Annette K. Brenner, Maria Hernandez-Valladares, Even Birkeland, Olav Mjaavatn, Håkon Reikvam, Frode Selheim, Frode S. Berven and Øystein Bruserud *

Table S1. Clinical and biological characteristics of the patients included in the study. The patients are listed according to the three subsets (yellow, brown and dark brown color, respectively) identified in Figure 2 in the article. CD34 positivity was defined as at least 20% positive cells in flow cytometric analysis. The three exceptional patients identified in Figure 1 (left part) of the article are marked with yellow in the Id column to the left in the table (patients 8, 9 and 10). The survival columns indicates (i) patients receiving intensive and potentially curative antileukemic treatment possibly including allogeneic stem cell transplantation; the survival is presented as months after start of therapy (yellow background; the sign > indicates that the patient is still alive without AML at the indicated time whereas the other patients are dead due to relapse unless otherwise stated); (ii) some of the elderly and unfit patients (indicated by green background, all patients surviving for less than 3 months) received low-toxicity AML-stabilizing therapy based on hydroxyurea, 5-mercaptopurine, low-dose cytarabine or azacitidine (survival in parenthesis); and (iii) the last group received only supportive care including antibiotic therapy and transfusions (blue background, all of them surviving less than 3 months).

Id	Gender	Age	Predisposition/previous hematological disease	FAB	Cytogenetics	FLT3 status	NPM1 status	CD34	Survival
1	M	41		M1	t(8;21) del(9) - 20 -22 -3	wt	wt	pos	>6 months
2	M	59	MDS	M5	del(20), +8	ITD	wt	pos	29 months
3	F	78		M1	Normal	ITD	ins	neg	AML stabilizing therapy (2 months)
4	M	42		M2	Normal	ITD	wt	Pos	>118 months
5	F	55		M2	Normal	ITD	ins	Neg	24 months
6	M	87	Chronic myelofibrosis	M1	del(20)	wt	wt	Pos	Supportive care
7	F	77		M1	Nt	nt	ins	Neg	Supportive care
8	M	78	CMML 9 months	M4	+8	nt	nt	Neg	Supportive care
9	M	79	MDS 2 years	M2	+8, -9	wt	wt	Pos	Supportive care
10	M	72	MDS	M4	Normal	nt	nt	Neg	Supportive care
11	M	20		M2	Normal	ITD	wt	Pos	27 months
12	M	71		M4/5	Nt	nt	nt	nt	AML stabilizing therapy (< 1 month)
13	F	68		M5	Normal	wt	ins	Neg	<1 month (toxic death)
14	F	87		M0	del(5)	wt	wt	Pos	AML stabilizing therapy (< 1 month)
15	F	63		M1	Normal	wt	wt	Pos	5 months
16	M	46		M1	Normal	wt	ins	Nt	26 months
17	M	36		M5	+8, +22, inv(16)	ITD	wt	Pos	27 months (toxic death in relapse)
18	M	78		M1	Multiple	nt	nt	Pos	Supportive care
19	F	77	MDS 2 years	M1	Normal	wt	wt	Pos	AML stabilizing therapy (1 month)

20	F	71	MDS, now AML relapse		del(12)	nt	nt	Pos	26 months
21	F	57		M4	inv16	wt	wt	Pos	14 months (denied further treatment)
22	F	79			Normal	ITD	ins	Pos	AML stabilizing therapy (2 months)
23	F	67	Relapsed AML	M0	+21	wt	wt	Pos	7 months
24	M	78		M1	Normal	wt	wt	pos	Supportive care
25	F	46		M4	Normal	ITD	Ins	pos	7 months
26	F	74	MDS	M5	Normal	ITD	ins	neg	Supportive care
27	F	71		M0	Normal	wt	ins	neg	Supportive care
28	M	82			+8	wt	wt	pos	Supportive care
29	F	77	MDS	M1/2	Normal	ITD	ins	nt	AML stabilizing therapy (1 month)
30	F	18		M4	Inv(16)	wt	wt	Pos	> 120 months
31	M	60		M5	t(10;11), +8	wt	wt	Nt	16 months
32	M	76	MDS 6 months		Normal	nt	nt	Pos	Supportive care
33	F	46		M2	inv(13)	wt	wt	Pos	29 months
34	F	55		M1	Normal	ITD	ins	Pos	8 months
35	M	76	MDS	M5	del(12), -7	nt	nt	Pos	AML stabilizing therapy (1 month)
36	F	66		M4/5	t(9;15)	ITD	wt	Neg	AML stabilizing therapy (2 month)
37	F	72	Relapsed AML	M2	Normal	ITD	ins	Pos	Supportive care
38	M	19		M5	Normal	wt	wt	Neg	> 66 months
39	M	48		M5	Normal	ITD	ins	Nt	7 months
40	M	64		M5	Normal	wt	ins	Neg	<1 month (toxic)
41	M	83		M1	Nt	wt	wt	Pos	Supportive care

Abbreviations: F, female; FAB, French-American-British; ITD, internal tandem duplication; ins, insertion; M, male; MDS, myelodysplastic syndrome; neg, negative; nt, not tested; pos, positive; wt, wild type.

Table S2. Proteomic analysis of MSC lysates derived from cells exposed to AML conditioned medium (i.e. AML cell culture supernatants); a comparison of the two patient clusters identified in Figure 2. AML cells were cultured for 48 hours before supernatants were harvested. Bone marrow MSCs derived from a healthy individual were cultured in the presence of AML supernatants (referred to as AML conditioned medium) for 48 hours before the MSCs proteome was analyzed. A hierarchical clustering analysis of the MSC proteome identified two cell patient subsets. The table presents those proteins that could be quantified for (i) at least five of the 10 patients in the upper yellow cluster but only three or less of the 31 patients in the brown lower cluster; or (ii) for at least 15 of the 31 patients in the brown cluster but for three or less patients in the yellow cluster). The table presents the gene name and the protein name, counts cluster 2 and counts cluster 1 represent the number of patients with quantified protein levels for each of the two main patient clusters identified in Figure 2, the Key words reflect main protein characteristics according to the Human Protein Atlas (The Human Protein Atlas accessed 30.07.2021). The Prognostic impact in cancer column to the right indicates whether a prognostic impact of the protein has been observed for any form of cancer according to the Human Protein Atlas. Red color column indicates higher levels in upper yellow main patent cluster that included 10 patients; blue color indicates higher levels in the lower brown main patient cluster 1 including 31 patients.

(yellow)	Intercellular communication, adhesion, extracellular matrix, receptor proteins	17
(green)	Golgi/endoplasmic reticulum/lysosome/vesicle proteins, cytoskeleton, intracellular transport	12
(blue)	Mitochondria, metabolism	5
(lilac)	RNA/DNA binding, protein synthesis	10

**Gene Symbol**	**Description**	**Cluster 2 (n=10); patient count**	**Cluster 1 (n=31); patient count**	**Key words**	**Prognostic impact in cancer**
**Common in the upper yellow cluster (at least 5 patients in the upper yellow cluster, three or less patients in the lower brown main cluster)**					
LAIR1	Leukocyte-associated immunoglobulin-like receptor 1	6	1	Plasma membrane receptor protein	A
RTN4	Isoform C of Reticulon-4	6	3	Endoplasmic reticulum, nuclear envelope, intracellular trafficking	A
CAMP	Cathelicidin antimicrobial peptide	5	2	Chemotactic protein	F
CYP4F3	Cytochrome P450 4F3	5	2	Endoplasmic reticulum, fatty acid metabolism	-
IGSF6	Immunoglobulin superfamily member 6	5	1	Cytoplasmic protein	F
IL3RA	Interleukin-3 receptor subunit alpha	5	2	IL3 receptor chain alpha	F
DEFA4	Neutrophil defensin 4	5	2	Secreted host defense protein	-
PGLYRP1	Peptidoglycan recognition protein 1	5	3	Pattern receptor, vesicles, nucleoplasm	-

PIK3R6	Phosphoinositide 3-kinase regulatory subunit 6	5	3	Signaling downstream to G-protein coupled receptors	A
PTGS2	Prostaglandin G/H synthase 2	5	3	Endoplasmic reticulum, fatty acid metabolism, dual cyclooxygenase and peroxidase	A
RETN	Resistin	5	3	Chemotaxis	-
SYTL1	Synaptotagmin-like protein 1	5	3	Vesicle trafficking, exocytosis	F/A
LAIR1	Leukocyte-associated immunoglobulin-like receptor 1	6	1	Plasma membrane, receptor protein	A
**Common in the lower brown main patient cluster (at least 15 patients in the lower brown main cluster, three or less patients in the upper yellow main cluster)**					
LTBP4	Latent-transforming growth factor beta-binding protein 4	2	24	TGF regulation	A
BCAM	Basal cell adhesion molecule	3	23	Laminin cell surface receptor	F/A
COL8A2	Collagen alpha-2(VIII) chain	3	23	Adhesion molecule	F/A
SGCG	Gamma-sarcoglycan	2	23	ECM-cytoskeleton linking	-
CD99	CD99 antigen	2	22	Cell adhesion and migration	A
FN1	Isoform 10 of Fibronectin	2	22	Cell adhesion	A
NUAK1	NUAK family SNF1-like kinase 1	1	22	Kinase that controls cell adhesion	A
AP5S1	AP-5 complex subunit sigma-1	3	21	Adaptor protein involved in endosomal transport	F
SLC39A6	Zinc transporter ZIP6 OS=Homo sapiens	3	20	Ion transport (probably Zn)	A
CPED1	Cadherin-like and PC-esterase domain-containing protein 1	2	20	Nucleoplasm	F/A
DTNBP1	Dysbindin OS=Homo sapiens OX=9606 GN=DTNBP1 PE=1 SV=1	2	20	Organelle biogenesis and vesicle trafficking.	F/A
FLNB	Filamin-B	1	20	Actinbinding, cytoskeleton	F/A
FOXO3	Forkhead box protein O3	3	19	Transcription factor	-
IDS	Iduronate 2-sulfatase	3	19	Sulfatase, lysosome	-
CCSER2	Serine-rich coiled-coil domain-containing protein 2	2	19	Nucleoplasm and cytosol, microtubule-binding	-
DKK3	Dickkopf-related protein 3	3	18	Regulation of Wnt signaling	-
EIF5A2	Eukaryotic translation initiation factor 5A-2	3	18	Elongation factor, RNA binding	A
GAP43	Neuromodulin	3	18	Calmodulin-binding	-
MYO6	Isoform 2 of Unconventional myosin-VI	1	18	Actin-binding, intracellular transport	F
EFHD1	EF-hand domain-containing protein D1	1	18	Calciumbinding, mitochondrial calcium sensor, cytoskeletal rearrangement	F

HSBP1	Heat shock factor-binding protein 1	1	18	Transcriptional regulation	F
MRFAP1	MORF4 family-associated protein 1	3	17	Nucleoplasm	F
SLC43A1	Large neutral amino acids transporter small subunit 3	3	17	Golgi, intracellular transport	F/A
ATN1	Atrophin-1	2	17	Nucleoplasm, transcriptional regulator	A
NCAM1	Isoform 2 of Neural cell adhesion molecule 1	2	17	Cell adhesion	F/A
HNRNPA3	Isoform 2 of Heterogeneous nuclear ribonucleoprotein A3	3	16	Nucleoplasm, RNA binding	F/A
LPL	Lipoprotein lipase	3	16	Triglyceride metabolism	A
SLC16A2	Monocarboxylate transporter 8	3	16	Intracellular transport	A
NDEL1	Nuclear distribution protein nudE-like 1	3	16	Cytosleleton, microtubules	-
OSBP2	Oxysterol-binding protein 2 OS=Homo sapiens	3	16	Lipid transport	F
PDE4DIP	Myomegalin OS=Homo sapiens	1	16	Golgi	-
MMP3	Stromelysin-1 OS=Homo sapiens	1	16	Degrades several extracellular matrix molecules	A
GDAP1	Ganglioside-induced differentiation-associated protein 1	3	15	Mitochondria	F/A
TTC31	Tetratricopeptide repeat protein 31	3	15	Cytosol and nucleoplasma	FA/A
ZNF574	Zinc finger protein 574	3	15	DNA binding, transcriptional regulation	-
POLR2M	DNA-directed RNA polymerase II subunit GRINL1A, isoforms 4/5	2	15	RNA polymerase	-
DSG2	Desmoglein-2 OS=Homo sapiens OX=9606 GN=DSG2 PE=1 SV=2	1	15	Cell adhesion, desmosome	F/A
RETN	Resistin	5	3	Chemotaxis	-
SYTL1	Synaptotagmin-like protein 1	5	3	Vesicle trafficking, exocytosis	F/A
LAIR1	Leukocyte-associated immunoglobulin-like receptor 1	6	1	Plasma membrane, receptor protein	A

Table S3. Proteomic analysis of AML cell supernatants, a comparison of the two patient clusters identified in Figure 2. AML cells were cultured for 48 hours before supernatants were harvested. Bone marrow MSCs derived from a healthy individual were cultured in the presence of AML supernatants (referred to as AML conditioned medium) for 48 hours before the MSCs proteome was analyzed. A hierarchical clustering analysis of the MSC proteome identified two cell patient subsets. The table presents those proteins that could be quantified for at least three patients in each of the two patients clusters/subsets and showed a statistically significant difference between the two groups (defined as a p-value <0.01 both for the t-test and for the Z-statistics of fold changes). The table presents the gene name and the protein name, counts cluster 2 and counts cluster 1 represent the number of patients with quantified protein levels for each of the two main patient clusters identified in Figure 2, the Key words reflect main protein characteristics according to the Human Protein Atlas (The Human Protein Atlas accessed 210730), and the Prognostic impact in cancer column to the right indicate whether a prognostic impact of the protein has been observed for any form of cancer according to the Human Protein Atlas. Red color of the gene name column indicates higher levels in the upper yellow patient cluster that included 10 patients; blue color indicates higher levels in the lower main patient cluster 1 including 31 patients.

(yellow)	Intercellular communication, adhesion, extracellular matrix	9 proteins
(green)	Golgi/endoplasmic reticulum/lysosome/vesicle proteins, cytoskeleton, intracellular transport	3 proteins
(blue)	Proteases, protease inhibitors	2 proteins
(lilac)	RNA/DNA binding, protein synthesis	4 proteins

**Gene name**	**Protein names**	**Cluster 2 (n=10); patient count**	**Cluster 1 (n=31); patient count**	**Key words**	**Prognostic impact in cancer**
RPN1	Dolichyl-diphosphooligosaccharide--protein glycosyltransferase subunit 1	7	7	Endoplasmic reticulum	A
IMMT	MICOS complex subunit MIC60	10	19	Mitochondria	F/A
HADHB	Trifunctional enzyme subunit beta, mitochondrial;3-ketoacyl-CoA thiolase	8	11	Mitochondria, lipid metabolism	F
MPO	Myeloperoxidase;Myeloperoxidase;89 kDa myeloperoxidase;84 kDa myeloperoxidase;Myeloperoxidase light chain;Myeloperoxidase heavy chain	10	30	Azurophilic granule	.
CYB5R3	NADH-cytochrome b5 reductase 3;NADH-cytochrome b5 reductase 3 membrane-bound form;NADH-cytochrome b5 reductase 3 soluble form	8	16	Endoplasmic reticulum, lipid metabolism	A
BANF1	Barrier-to-autointegration factor;Barrier-to-autointegration factor, N-terminally processed	10	30	DNA binding, chromatin organization	-
RPS19	40S ribosomal protein S19	7	29	Ribosomal protein	A
TGM2	Protein-glutamine gamma-glutamyltransferase 2	8	18	Plasma membrane, protein crosslinking	A

SRSF3	Serine/arginine-rich splicing factor 3	5	21	Nucleosome, RNA splicing	A/F
EPRS	Bifunctional glutamate/proline--tRNA ligase;Glutamate--tRNA ligase;Proline--tRNA ligase	10	27	RNA binding, protein syntesis	A
PUF60	Poly(U)-binding-splicing factor PUF60	7	25	RNA processing/splicing	-
MFGE8	Lactadherin;Lactadherin short form;Medin	5	20	Secreted membrane protein, implicated in angiogenesis and carcinogenesis.	A
COL5A2	Collagen alpha-2(V) chain	10	30	Extracellular matrix	A
PAPPA	Pappalysin-1	6	26	Secreted metalloprotease, IGF regulator	-
IGFBP7	Insulin-like growth factor-binding protein 7	10	30	Low-affinity IGF binding, regulates IGF availability and involved in cell adhesion.	F/A
QSOX1	Sulfhydryl oxidase 1	10	30	Oxidoreductase, located to Golgi and vesicles, secreted protein.	A
C1R	Complement C1r subcomponent;Complement C1r subcomponent heavy chain;Complement C1r subcomponent light chain	6	28	Complement activation, protease	A
ICAM1	Intercellular adhesion molecule 1	3	12	Adhesion molecule	F/A
MMP19	Matrix metalloproteinase-19	4	12	Matrix metalloprotease, degrades various extracellular matrix molecules	F/A
EMILIN2	EMILIN-2	4	12	Extracellular matrix protein	A
DCN	Decorin	9	30	Extracellular matrix molecule	A
SERPINE2	Glia-derived nexin	10	30	Protease inhibitor	A

Table S4. Proteomic analysis of AML cell supernatants, a comparison of the two patient clusters identified in Figure 2. AML cells were cultured for 48 hours before supernatants were harvested. Bone marrow MSCs derived from a healthy individual were cultured in the presence of AML supernatants (referred to as AML conditioned medium) for 48 hours before the MSCs proteome was analyzed. A hierarchical clustering analysis of the MSC proteome identified two cell patient subsets. The table presents those proteins that could be either (i) for at least five patients in the smaller upper/yellow cluster 2 but three or less patients in the larger main lower/brown cluster; or (ii) at least 15 patients in the larger/brown main patient cluster but three or less patients in the smalle upper/yellow cluster. The table presents the gene name and the protein name, counts cluster 2 and counts cluster 1 represent the number of patients with quantified protein levels for each of the two main patient clusters identified in Figure 2, the Key words reflect main protein characteristics according to the Human Protein Atlas (The Human Protein Atlas accessed 30.07.21), and the Prognostic impact in cancer column to the right indicate wheter a prognostic impact of the protein has been observed for any form of cancer according to the Human Protein Atlas.

(yellow)	Intercellular communication, adhesion, extracellular matrix	22 proteins
(green)	Golgi/endoplasmic reticulum/lysosome/vesicle proteins, cytoskeleton, intracellular transport	22 proteins
(blue)	Proteases, protease inhibitors	2 proteins
(lilac)	Intracellular signaling	4 proteins

Gene name	Protein names	Cluster 2 (n=10); patient count	Cluster 1 (n=31); patient count	Key words	Prognostic impact in cancer
**Common in the upper yellow main patient cluster (at least 5 patients of the 10 patients in this cluster, only three or less patients in the lower brown main cluster)**					
MAP2K1	Dual specificity mitogen-activated protein kinase kinase 1	6	1	Cell signaling, MAP kinase pathway	A
UNC13D	Protein unc-13 homolog D	6	3	Exocytosis, vesicle formation	A
ZPR1	Zinc finger protein ZPR1	5	3	Vesicular protein, communicates signals from cytoplasm to nucleus.	A
ATP5H	ATP synthase subunit d, mitochondrial	5	3	Intracellular ion transport, mitochondrial	-
ERLIN1	Erlin-1	5	2	Endoplasmic reticulum protein, cholesterol binding.	A
M6PR	Cation-dependent mannose-6-phosphate receptor	5	1	Intracellular transport (from Golgi to lysosomes)	A
MOB4	MOB-like protein phocein	5	3	Intracellular transport	A
TMX4	Thioredoxin-related transmembrane protein 4	5	3	Endoplasmic reticulum, protein folding	F
THOC7	THO complex subunit 7 homolog	5	2	RNA transport and processing	-

TNC	Tenascin	5	0	Cell adhesion, extracellular matrix protein	-
**Common in the lower brown main cluster 1 (quantified for at least 15 patients in the lower cluster, only three or less patients in the yellow upper main cluster)**					
SERPING1	Plasma protease C1 inhibitor	3	26	Protease inhibitor, regulates complement and coagulation activation	F/A
GAA	Lysosomal alpha-glucosidase;76 kDa lysosomal alpha-glucosidase;70 kDa lysosomal alpha-glucosidase	3	24	Lysosomal protein	A
MET	Hepatocyte growth factor receptor	3	24	HGF cell surface receptor (protooncogene)	A
POSTN	Periostin	3	24	Cell adhesion, extracellular matrix protein	A
SPN	Leukosialin	3	24	Cell surface glycoprotein	A
DES	Desmin	3	24	Cytoskeleton-microtubules	A
MANBA	Beta-mannosidase	3	23	Lysosomal protein	A
PFDN2	Prefoldin subunit 2	3	22	Chaperon	A
PPP1R2;PPP1R2P3	Protein phosphatase inhibitor 2;Protein phosphatase inhibitor 2-like protein 3	3	22	Protein phosphatase 1 inhibitor. Cell signaling	-
QPCT	Glutaminyl-peptide cyclotransferase	2	22	Cytosolic acyltransferase	-
SELL	L-selectin	2	21	Adhesion molecule, plasma membrane	F/A
VCAM1	Vascular cell adhesion protein 1	3	21	Adhesion molecule, plasma membrane	F
FBN2	Fibrillin-2	3	20	Extracellular matrix molecule	A
FNDC1	Fibronectin type III domain-containing protein 1	2	20	Secreted to extracellular matrix	F/A
ITGB2	Integrin beta-2	3	20	Adhesion molecule, plasma membrane	-
PEA15	Astrocytic phosphoprotein PEA-15	3	20	Intracellular signaling; integrin and MAP kinase signaling.	A
AP3D1	AP-3 complex subunit delta-1	2	19	Intracellular transport, Golgi to lysosomes.	F/A
C1orf123	UPF0587 protein C1orf123	3	19	Located to vesicles and mitotic spindles.	A
CLIP1	CAP-Gly domain-containing linker protein 1	3	19	Microtubile cytoskeleton, vesicle linking	-
COL6A2	Collagen alpha-2(VI) chain	3	19	Extracellular matrix	F/A
ARHGAP4	Rho GTPase-activating protein 4	3	19	Intracellular signaling, Rho GTPases.	A
LOXL3	Lysyl oxidase homolog 3	3	19	Lipoxygenase, fatty acid metabolism	A
DDAH1	N(G),N(G)-dimethylarginine dimethylaminohydrolase 1	3	18	Hydrolase, nitric oxide generation.	F
ICAM2	Intercellular adhesion molecule 2	3	18	Plasma membrane, adhesion molecule	-
NCF1;NCF1B;NCF1C	Neutrophil cytosol factor 1;Putative neutrophil cytosol factor 1B;Putative neutrophil cytosol factor 1C	3	18	Oxidase, superoxide production	-

PLP2	Proteolipid protein 2	3	18	Endoplasmic reticulum, ion channel?	A
POSTN	Periostin	3	18	Cell adhesion, extracellular matrix protein	A
PPT1	Palmitoyl-protein thioesterase 1	2	18	Hydrolase, Golgi-vesicles.	A
LEPRE1	Prolyl 3-hydroxylase 1	3	18	Vesicle protein, extracellular matrix modulation	A
RPL15	60S ribosomal protein L15	3	18	Ribosome	-
ANGPTL4	Angiopoietin-related protein 4	3	18	Angioregulatory protein, secreted vesicle protein, trglyceride matbolism	A
ACP1	Low molecular weight phosphotyrosine protein phosphatase	3	17	Hydrolase, protein phosphatase	A
BLVRA	Biliverdin reductase A	3	17	Oxidoreductase	F
DNPH1	2-deoxynucleoside 5-phosphate N-hydrolase 1	3	17	Nucleotide metabolism	-
FAM134A	Protein FAM134A	3	17	Cytosolic	A
HBD	Hemoglobin subunit delta	3	17	Oxygen binding	-
HDHD1	Pseudouridine-5-phosphatase	3	17	Hydrolase, nucleotide metabolism	F/A
LRRC15	Leucine-rich repeat-containing protein 15	3	17		
MAN1B1	Endoplasmic reticulum mannosyl-oligosaccharide 1,2-alpha-mannosidase	3	17	Endoplasmic eticulum, chaperone regulator	A
PTPN12	Tyrosine-protein phosphatase non-receptor type 12	3	17	Protein phosphatase, plasma membrane	A
SRP68	Signal recognition particle subunit SRP68	3	17	Protein targeting to endoplasmic reticulum	F/A
COL5A3	Collagen alpha-3(V) chain	1	16	Extracellular matrix	A
CXCL8	Interleukin-8;MDNCF-a;Interleukin-8;IL-8(5-77);IL-8(6-77);IL-8(7-77);IL-8(8-77);IL-8(9-77)	2	16	Angioregulatory chemokine	A
ENG	Endoglin	1	16	Cell adhesion, cytokine signaling	F/A
HIST2H2BE	Histone H2B type 2-E;Histone H2B type 1-B;Histone H2B type 1-O;Histone H2B type 3-B	3	16	Nucleosome	A
LRMP	Lymphoid-restricted membrane protein;Processed lymphoid-restricted membrane protein	3	16	Endoplasmic reticulum, peptide transport	-
SFN	14-3-3 protein sigma	3	16	Intracellular signaling	F/A
TIPRL	TIP41-like protein	3	16	Vesicular protein, phosphatase regulator	A
VDAC1	Voltage-dependent anion-selective channel protein 1	2	16	Plasma and mitochondrial membrane channel	A
VNN1	Pantetheinase	3	16	Cell surface	A
VWF	von Willebrand factor;von Willebrand antigen 2	3	16	Coagulation, adhesion	A
ARF4	ADP-ribosylation factor 4	3	15	Intracellular transport	A

CD33	Myeloid cell surface antigen CD33	3	15	Cell adhesion	-
CHST11	Carbohydrate sulfotransferase 11	0	15	Sulfate transferase, Golgi	A
EIF3E	Eukaryotic translation initiation factor 3 subunit E	3	15	Protein synthesis	A
FKBP15	FK506-binding protein 15	3	15	Actin binding, cytoskeleton	A
GART	Trifunctional purine biosynthetic protein adenosine-3;Phosphoribosylamine--glycine ligase;Phosphoribosylformylglycinamidine cyclo-ligase;Phosphoribosylglycinamide formyltransferase	3	15	Purine biosynthesis	A
GSPT1;GSPT2	Eukaryotic peptide chain release factor GTP-binding subunit ERF3A;Eukaryotic peptide chain release factor GTP-binding subunit ERF3B	3	15	Protein synthesis	F/A
IK	Protein Red	2	15	RNA splicing	F/A
LAMP1	Lysosome-associated membrane glycoprotein 1	3	15	Cell membrane glycoprotein, selectin modulation	F
NEK9	Serine/threonine-protein kinase Nek9	3	15	Kinase, cell cycle regulation	F
PARVG	Gamma-parvin	3	15	Cell adhesion, cytoskeleton	F/A
PROSC	Proline synthase co-transcribed bacterial homolog protein	2	15		-
RPL14	60S ribosomal protein L14	3	15	Ribosome	-
TFPI	Tissue factor pathway inhibitor	3	15	Coagulation, protease inhibitor	A
MMP10	Stromelysin-2	3	15	Protease, collagen degradation	F

Table S5. Proteomic analysis of the extracellular AML cell secretome; a comparison of the two main clusters identified in Figure 2 in the article. This table presents a more detailed classification of the 44 proteins from Table S4 that are involved in cellular communication and intracellular organelles/trafficking/transport. The gene names are used for identification of proteins; underlined names refer to proteins increased in the upper yellow main patient cluster (including 10 patients) whereas the other proteins are increased in the lower brown main patient cluster (31 patients). The classification is based on the Protein Atlas database (The Human Protein Atlas accessed 29.07.2021).

**CELLULAR COMMUNICATION**	
	**Cell surface/plasma membrane molecules:** MET, SPN, SELL, VCAM1, PTPN12, VNN1
	**Cell adhesion:** TNC, POSTN, SELL, VCAM1, ICAM2, COL5A, ENG, VWF, CD33,
	**Extracellular matrix (including matrix modulators):** TNC, POSTN, FBN2, FNDC1, ITGB2, COL6A2, LEPRE1, LAMP1, MMP10
	**Other proteins involved in cellular communication:** ANGPTL4, CXCL8, VWF
**INTRACELLULAR TRANSPORT/TRAFFICKING:**	
	**Vesicular proteins:** UNC13D, C1orf123, PPT1, LEPRE1, TIPRL
	**Endoplasmic reticulum:** ERLIN1, TMX4, PLP2, MAN1B1, SRP68, SRP68, LNRP
	**Golgi:** CHST11
	**Lysosomes:** GAA, MANBA,
	**Cytoskeleton:** DES, CLIP1, FKBP15, PARVG
	**Intracellular transport:** M6PR, MOB4, THOC7, AP3D1, ARF4

Table S6. Proteins detected only in MSCs cultured with AML-conditioned medium (i.e. AML induced MSC proteins) but not in control MSCs cultured in medium alone. The table shows the MSC proteins detected for AML conditioned medium derived from at least 30 of the 41 patients, and for each protein we give the gene name together with the number of patient samples resulting in detectable levels in parenthesis, a short description of the protein and key words. The information is based on the Gene database, selected references from the PubMed database (PubMed (nih.gov) accessed 29.07.21) and the Human Protein Atlas (The Human Protein Atlas accessed 29.07.2021).

**Gene (number of patients)**	**Description**	**Key words**
VAV1 (40)	*Vav guanine nucleotide exchange factor 1.* This protein is a member of the VAV gene family, a family of guanine nucleotide exchange factors (GEFs) for Rho family GTPases that activate pathways leading to actin cytoskeletal rearrangements and transcriptional alterations. The encoded protein is important in hematopoiesis.	Rho family<br>Cytoskeleton<br>Transcription
BIN2 (40)	*Bridging integrator 2.* The protein has a broad expression in the bone marrow and is associated with actin-rich structures of the plasma membrane; it is involved in cell migration and possibly intracellular transport/endocytosis (PMID 23285027). The Stem Cell Factor (SCF) maintains stemness of MSCs by maintaining mitochondrial functions (PMID 32736659); this effect is mediated through various pathways and BIN2 is one of the downstream target of SCF signaling (PMID 23185384).	Actin<br>Itracellular transport<br>Stemness
NBEAL2 (39)	*Neurobeachin-like protein 2.* The protein contains a beige and Chediak-Higashi (BEACH) domain and multiple WD40 domains, and may play a role in megakaryocyte alpha-granule biogenesis. The gene shows only low expression in various mesenchymal cells (Cell atlas - NBEAL2 - The Human Protein Atlas)	Granule formation
TLR2 (38)	*Toll-like receptor 2.* The protein is a member of the Toll-like receptor (TLR) family. TLR2 is a cell-surface protein that can form heterodimers with other TLR family members to recognize conserved exogenous or endogenous ligands Activation of TLRs leads to an up-regulation of signaling pathways including NFκB.	NFκB
GZMK (38)	*Granzyme K.* This protein is a member of a group of related serine proteases from the cytoplasmic granules of cytotoxic cells. The protein described here lacks consensus sequences for N-glycosylation present in other granzymes. Low expression has previously been detected in a sarcoma cell line (Cell atlas - GZMK - The Human Protein Atlas).	Protease
CD84 (38)	*CD84 molecule.* The encoded membrane glycoprotein is a member of the signaling lymphocyte activation molecule (SLAM) family. The encoded protein is a homophilic adhesion molecule that is involved in regulating receptor-mediated signaling. The protein is expressed in a wide range of organs, but with very low or absent expression in normal mesenchymal cells (Cell type atlas - CD84 - The Human Protein Atlas)	Adhesion<br>Signal transduction
UNC13D (37)	*Unc-13 homolog.* This protein is a member of the UNC13 family, containing similar domain structure as other family members but lacking an N-terminal phorbol ester-binding C1 domain present in other Munc13 proteins. The protein appears to play a role in vesicle maturation during exocytosis.	Vesicle maturation<br>Exocytosis
CFD (37)	*Complement factor D.* This gene encodes a member of the chymotrypsin family of serine peptidases. This protease catalyzes the cleavage of factor B, the rate-limiting step of the alternative pathway of complement activation. This protein also functions as a cell-signaling adipokine. The protein can be released by fibroblasts and functions as a modulator of the extracellular matrix (PMID 31026383).	Protease<br>Extracellular matrix
ELMO1 (37)	*Engulfment and cell motility protein 1.* This gene encodes a member of the engulfment and cell motility protein family. These proteins interact with dedicator of cytokinesis proteins to promote phagocytosis and cell migration. The protein can be expressed at low levels in	Phagocytosis

	mesenchymal cells ([Cell atlas - ELMO1 - The Human Protein Atlas]).	
GP9 (37)	*Platelet glycoprotein IX.* This gene encodes a small membrane glycoprotein. It forms a 1-to-1 noncovalent complex with glycoprotein Ib, a platelet surface membrane glycoprotein complex that functions as a receptor for von Willebrand factor.	Cell membrane glycoprotein
IRAK3 (37)	*Interleukin-1 receptor-associated kinase 3.* The encoded protein is a member of the interleukin-1 receptor-associated kinase protein family. Members of this family are essential components of the Toll/IL-R immune signal transduction pathways. It functions as a regulator of Toll-like receptor signaling.	IL1 receptor TLR
LYN (36)	*Tyrosine-protein kinase Lyn.* This gene encodes a tyrosine protein kinase that may be involved in the regulation of mast cell degranulation and erythroid differentiation.	Tyrosine kinase Degranulation
ICAM3 (36)	*Intercellular adhesion molecule 3.* The protein encoded by this gene is a member of the intercellular adhesion molecule (ICAM) family of transmembrane glycoproteins that bind to the leukocyte adhesion LFA-1 protein. It functions not only as an adhesion molecule, but also as a potent signaling molecule.	Adhesion Signaling
HLA-DRB1 (35)	HLA class II histocompatibility antigen, DRB1 beta chain.	HLA molecule
CXCL7/PPBP (35)	*CXCL7 (Platelet basic protein).* The protein encoded by this gene is a platelet-derived growth factor that belongs to the CXC chemokine family. This growth factor is a potent chemoattractant and activator of neutrophils. It has been shown to stimulate various cellular processes including DNA synthesis, mitosis, glycolysis, intracellular cAMP accumulation, prostaglandin E2 secretion, and synthesis of hyaluronic acid and sulfated glycosaminoglycan. It binds to G-protein coupled receptors that also can be expressed by MSCs and autocrine stimulation may thereby be possible (PMID 18793669, 33865426).	Chemokine G protein Autocrine
AGTPBP1 (35)	*Cytosolic carboxypeptidase 1.* NNA1 is a zinc carboxypeptidase that contains nuclear localization signals and an ATP/GTP-binding motif. The protein can be expressed by various mesenchymal cells ([Cell atlas - AGTPBP1 - The Human Protein Atlas])	ATP/GTP
RAB37 (34)	*Ras-related protein Rab-37.* Rab proteins are low molecular mass GTPases that are critical regulators of vesicle trafficking. For additional background information on Rab proteins, see MIM 179508	GTPase Vesicle trafficking
DEFA1 (34)	*Neutrophil defensin 1.* Defensins are expressed in different tissues. Defensin, alpha 1 plays a role in phagocyte-mediated host defense. This protein is also a part of the MSC extracellular vesicle proteome (PMID 30378105).	Phagocytosis MSC vesicles
PLCB2 (34)	*1-phosphatidylinositol 4,5-bisphosphate phosphodiesterase beta-2.* The protein is a phosphodiesterase that catalyzes the hydrolysis of phosphatidylinositol 4,5-bisphosphate to the second messengers inositol 1,4,5-trisphosphate (IP3) and diacylglycerol. The encoded protein is activated by G proteins (i.e. a GTP binding protein). NFκB regulates the transcription of this gene.	Phosphodiesterase G protein NFκB
ARHGAP25 (33)	*Isoform 4 of Rho GTPase-activating protein 25.* ARHGAPs, such as ARHGAP25, encode negative regulators of Rho GTPases that are implicated in actin remodeling, cell polarity, and cell migration (PubMed 15254788). It is regarded as a Rac GTPase activating protein (PMID 27566826), and the RhoE/ROCK/ARHGAP25 signaling pathway is regarded as a Rac regulator (PMID 27413008). Rac/Rho GTPases are important regulators of microtubule organization and intracellular vesicular transport (PMID 25054920, 31358621) .	Rho GTPase Vesicle trafficking
ARHGAP45 (33)	Rho GTPase-activating protein 45. ArhGAP45 acts as a Rac-GAP (GTPase-Activating Protein) (PMID 29174013).	Rho GTPase Vesicle trafficking
GIMAP8 (33)	*GTPase IMAP family member 8.* This gene encodes a protein belonging to the GTP-binding superfamily and to the immuno-associated nucleotide (IAN) subfamily of nucleotide-binding proteins.	GTPase
BTNL8 (33)	*Butyrophilin-like protein 8.* The protein is predicted to be a membrane protein ([Cell atlas - BTNL8 - The Human Protein Atlas]).	

SASH3 (32)	*SAM and SH3 domain-containing protein 3.* The protein has a Src homology-3 (SH3) domain and a sterile alpha motif (SAM), both of which are found in proteins involved in cell signaling.	Cell signaling?
ARHGAP15 (31)	Rho GTPase-activating protein 15. RHO GTPases regulate diverse biologic processes, and their activity is regulated by RHO GTPase-activating proteins (GAPs), such as ARHGAP15 (PubMed 12650940). The protein shows nucleotide-independent Rac1 binding and induces actin remodeling.	Rho GTPase<br>Vesicle trafficking<br>Actin
CD36 30)	*Platelet glycoprotein 4.* The protein serves as a receptor for thrombospondin and functions as a cell adhesion molecule. It binds to collagen, thrombospondin, anionic phospholipids and oxidized LDL. It binds long chain fatty acids and may function in the transport and/or as a regulator of fatty acid transport. CD36 shows a vesicle-mediated reversible trafficking (recycling) between intracellular membrane compartments and the cell surface (PMID 27615427). Thrombospondin is also important for stem cell maintenance and extracellular **vesicle** functions (PMID 33925464),	Adhesion<br>Thrombospondin<br>Vesicle<br>Stemness
GIMAP1 (30)	*GTPase IMAP family member 1.* This protein belongs to the GTP-binding superfamily and to the immuno-associated nucleotide (IAN) subfamily of nucleotide-binding proteins.	GTP binding
APOBEC3F (30)	*Apolipoprotein B mRNA editing enzyme catalytic subunit 3F.* This gene is a member of the cytidine deaminase gene family. Members of the cluster encode proteins that are structurally and functionally related to the C to U RNA-editing cytidine deaminase APOBEC1. It is thought that the proteins may be RNA editing enzymes and have roles in growth or cell cycle control.	RNA<br>Growth control
DOK2 (30)	*Docking protein 2.* Downstream of kinase (Dok)-related protein (DokR is implicated in cytokine signaling, and tyrosine phosphorylation induces DokR to bind the signal relay molecule RasGTPase-activating protein (RasGAP).	GTPase
NSMCE3 (30)	*NSE3 homolog, SMC5-SMC6 complex component.* The protein encoded by this gene is part of the SMC5-6 chromatin reorganizing complex and is a member of the MAGE superfamily.	Chromatin

Table S7. The number of AML-induced MSC proteins for individual leukemia patients. We identified 301 proteins that could be quantified onlywhen MSCs had been cultured with AML conditioned medium (CM). The table presents the number of such AML induced proteins detected for each individual patient. The 10 samples belonging to the upper yellow samples cluster identified in Figure 2 in the article are marked with yellow (patients 1-10). The patients are numbered according to Table S1.

Culture condition	Number of proteins
MSC+P8-CM	174
MSC+P4-CM	149
MSC+P10-CM	149
MSC+P3-CM	147
MSC+P9-CM	145
MSC+P32-CM	131
MSC+P39-CM	123
MSC+P41-CM	123
MSC+P2-CM	110
MSC+P1-CM	106
MSC+P6-CM	106
MSC+P36-CM	105
MSC+P28-CM	104
MSC+P40-CM	102
MSC+P16-CM	98
MSC+P22-CM	98
MSC+P38-CM	98
MSC+P30-CM	95
MSC+P18-CM	90
MSC+P31-CM	90
MSC+P7-CM	90
MSC+P13-CM	88
MSC+P37-CM	85
MSC+P19-CM	83
MSC+P11-CM	81
MSC+P17-CM	81
MSC+P5-CM	78
MSC+P25-CM	77
MSC+P12-CM	75
MSC+P26-CM	70
MSC+P21-CM	67
MSC+P35-CM	67
MSC+P34-CM	67
MSC+P14-CM	66
MSC+P29-CM	64
MSC+P33-CM	60
MSC+P27-CM	50
MSC+P23-CM	48
MSC+P15-CM	47
MSC+P20-CM	42
MSC+P24-CM	34

Table S8. Protein enrichment classification of MSC proteins that are quantified both for MSCs cultured with AML conditioned medium and in MSC medium controls (at least three of the four controls), but showing significant differences in protein abundance between these two MSC groups. All identified proteins could be quantified for at least 24 of the 41 MSC samples that had been exposed to AML conditioned medium. The proteins were classified using a GO tool. The most significant general GO terms are listed together with foreground and background counts, s- and p-values and the false discovery rate.

Term	Description	Category	Fore-ground count	Back-ground count	s-value	p-value	FDR
**UPREGULATED**							
GO:0031410	Cytoplasmic vesicle	CC	56	1380	1.07	1.69×10^{-06}	0.00469
GO:0005615	Extracellular space	CC	60	1655	0.92	4.84×10^{-06}	0.00671
GO:0005576	Extracellular region	CC	65	1884	0.89	7.91×10^{-06}	0.00731
GOCC:0031982	Vesicle	CC-TM	56	1388	1.05	1.86×10^{-06}	0.00104
GOCC:0030141	Secretory granule	CC-TM	35	497	1.03	7.10×10^{-07}	0.00104
GOCC:0099503	Secretory vesicle	CC-TM	35	523	1.02	5.90×10^{-07}	0.00104
GOCC:0031410	Cytoplasmic vesicle	CC-TM	49	1167	0.92	3.21×10^{-06}	0.00104
GOCC:0031224	Intrinsic component of membrane	CC-TM	34	599	0.88	9.13×10^{-07}	0.00104
GOCC:0005615	Extracellular space	CC-TM	32	516	0.85	1.31×10^{-06}	0.00104
GOCC:0016021	Integral component of membrane	CC-TM	31	522	0.79	1.58×10^{-06}	0.00104
GOCC:0005576	Extracellular region	CC-TM	39	880	0.72	7.27×10^{-06}	0.00178
**DOWN-REGULATED**							
GO:0030198	Extracellular matrix organization	BP	17	202	1.87	4.03×10^{-07}	0.00802
GO:0030312	External encapsulating structure	CC	16	228	1.85	1.38×10^{-07}	0.00038
GO:0031012	Extracellular matrix	CC	16	228	1.85	1.38×10^{-07}	0.00038
GOCC:0005576	Extracellular region	CC-TM	22	880	1.82	5.67×10^{-07}	0.00111
GOCC:0031012	Extracellular matrix	CC-TM	14	355	1.41	2.65×10^{-07}	0.00104
GOCC:0062023	Collagen-containing extracellular matrix	CC-TM	8	103	0.81	1.25×10^{-06}	0.00163

S-value is a combination of (minus log) p-value and effect size (i.e. positive associations in the foreground divided by all associations); a positive value indicates overrepresentation of a given term, and a negative value indicates underrepresentation of a given term. Foreground counts indicate the number of positive associations for a given term (i.e. the number of proteins associated with the given term) and background counts indicate the number of positive association in the dataset.

Abbreviations: BP, GO biological process; CC, GO Cellular component TextMining; FDR, false discovery rate.

Table S9. Classification of MSC proteins that are quantified both for MSCs cultured with AML conditioned medium (at least 24 AML-affected MSC samples showing detectable levels) and in MSC medium controls (at least three of the four controls), but showing significant differences in protein abundance between these two MSC groups. The table presents the results for the four networks/subclusters presented in Figure 5. The proteins were classified using a GO tool. The most significant general GO terms and/or KEGG pathways are listed together with foreground and background counts, s- and p-values and the false discovery rate.

Term	Description	Category	Foreground count	Background count	s-value	p-value	FDR
**CLUSTER/NETWORK 1**							
GOCC:0030141	Secretory granule	CC-TM	27	497	6.41	1.30×10^{-07}	0.00022
GOCC:0005615	Extracellular space	CC-TM	16	516	3.54	1.54×10^{-07}	0.00022
GOCC:0042581	Specific granule	CC-TM	16	132	3.48	8.76×10^{-07}	0.00022
GO:0016192	Vesicle-mediated transport	BP	27	1226	5.60	1.75×10^{-07}	0.00324
GO:0002376	Immune system process	BP	27	1287	5.28	1.75×10^{-07}	0.00324
**CLUSTER/NETWORK 2**							
map04514	Cell adhesion molecules	KEGG	6	57	5.71	1.89×10^{-07}	1.37×10^{-05}
**CLUSTER/NETWORK 3**							
map03030	DNA replication	KEGG	5	29	5.98	9.80×10^{-07}	0.00021
map04110	Cell cycle	KEGG	4	65	5.85	3.96×10^{-08}	1.73×10^{-05}
**CLUSTER/NETWORK 4**							
GOCC:0031093	Platelet alpha granule lumen	CC-TM	4	49	3.32	1.85×10^{-07}	0.00073
GOCC:0070013	Intracellular organelle lumen	CC-TM	8	2208	2.81	8.50×10^{-05}	0.039
GOCC:0005576	Extracellular region	CC-TM	6	880	2.57	8.03×10^{-05}	0.039

S-value is a combination of (minus log) p value and effect size (i.e. positive associations in the foreground divided by all associations); a positive value indicates overrepresentation of a given term, and a negative value indicates underrepresentation of a given term. Foreground counts indicate the number of positive associations for a given term (i.e. the number of proteins associated with the given term) and background counts indicate the number of positive association in the dataset.
Abbreviations: BP, GO biological process; CC-TM, GO Cellular component TextMining; FDR, false discovery rate; KEGG, KEGG pathway.

Table S10. Proteins quantified in control MSCs but only in 10 or less of the 41 MSC samples exposed to AML conditioned medium. The 27 individual proteins listed in the table were quantified either in at least three of the four MSC control samples (the three upper proteins, indicated by blue color) or in at least one control MSC sample (the 24 other proteins, indicated by green color). The table presents the identity of the protein (i.e. the gene name, the number of AML exposed MSCs with quantifiable level is indicated in parenthesis), a brief description of the protein is given together with key words reflecting important functions. The description is based on the Human Protein Atlas (The Human Protein Atlas accessed 05.10.2021).

Gene Symbol	Description	Key words
KTN1 (5)	*Kinectin 1.* This gene encodes an integral membrane protein that is a member of the kinectin protein family. The encoded protein is primarily localized to the endoplasmic reticulum membrane. This protein binds kinesin and may be involved in intracellular organelle motility. This protein also binds translation elongation factor-delta and may be involved in the assembly of the elongation factor-1 complex.	Endoplasmic reticulum<br>Intracellular transport<br>Protein synthesis
EIF1B (8)	*Eukaryotic translation initiation factor 1B.* The protein is probably involved in translation and protein synthesis.	Protein synthesis
COL6A3 (9)	*Collagen type VI alpha 3 chain.* The encoded protein is found in most connective tissues. The alpha-3 chain of type VI collagen is much larger than the alpha-1 and -2 chains. This difference in size is largely due to an increase in the number of subdomains, similar to von Willebrand Factor type A domains, which are found in the amino terminal globular domain of all the alpha chains. These domains have been shown to bind extracellular matrix proteins, an interaction that explains the importance of this collagen in organizing matrix components.	Extracellular matrix
CAMKK2 (2)	*Calcium/calmodulin-dependent protein kinase 2.* The encoded protein belongs to the Serine/Threonine protein kinase family, and to the Ca^{++}/calmodulin-dependent protein kinase subfamily. The major isoform of this gene plays a role in the calcium/calmodulin-dependent (CaM) kinase cascade by phosphorylating the downstream kinases CaMK1 and CaMK4. Protein products of this gene also phosphorylate AMP-activated protein kinase (AMPK).	Ser/Thr protein kinase
POSTN (3)	*Periostin.* This gene encodes a secreted extracellular matrix protein that binds to integrins to support adhesion and migration of cells. This protein plays a role in cancer stem cell maintenance and metastasis.	Extracellular matrix<br>Adhesion, migration
KPRP (5)	*Keratinocyte proline-rich protein.* The gene encodes an intracellular protein.	
BUD13 (5)	*BUD13 homolog.* The encoded nucleoplasmic protein is involved in mRNA processing and splicing.	RNA processing
CHIT1 (5)	*Chitotriosidase-1/chitinase-1.* This secreted protein is involved in carbohydrate metabolism and polysaccharide degradation.	Polysaccharide degradation
PIP (5)	*Prolactin-inducible protein.* This gene encodes a secreted actin-binding protein.	Actin
SERPINB12 (5)	*Serpin B12 (serpin family B member 12).* The encoded protein is a serine proteinase inhibitor.	Protease inhibitor
ERCC6L (6)	*DNA excision repair protein ERCC-6-like.* The protein is a member of the SWItch/Sucrose Non-Fermentable (SWI/SNF2) family of proteins, and during interphase the protein is excluded from the nucleus, and only associates with chromatin after the nuclear envelope has broken down. This protein is a DNA translocase that is thought to bind double-stranded DNA. The protein also associates with ribosomal DNA and ultra-fine DNA bridges (UFBs), fine structures that connect sister chromatids during	DNA translocase

	anaphase.	
FOLR3 (6)	*Folate receptor gamma.* This gene encodes a member of the folate receptor family of proteins, which have a high affinity for folic acid and for several reduced folic acid derivatives. It mediates delivery of 5-methyltetrahydrofolate to the interior of cells.	Folic acid
MBD1 (6)	*Methyl-CpG-binding domain protein 1.* The protein is a DNA binding transcriptional regulator.	DNA binding
MCC (7)	*Colorectal mutant cancer protein.* This intracellular protein is involved in Wnt signaling.	Wnt signaling
ACOX1 (7)	*Acyl-coenzyme A oxidase 1.* The protein is the first enzyme of the fatty acid beta-oxidation pathway, which catalyzes the desaturation of acyl-CoAs to 2-trans-enoyl-CoAs. It donates electrons directly to molecular oxygen, thereby producing hydrogen peroxide.	Fatty acid metabolism
S100P (7)	*Protein S100-P.* The protein is a member of the S100 family of proteins containing 2 EF-hand calcium-binding motifs. S100 proteins are localized in the cytoplasm and/or nucleus and are involved in a number of cellular processes such as cell cycle progression and differentiation. This protein, in addition to binding Ca2+, also binds Zn2+ and Mg2+.	Cell cycle regulation
MINDY1 (7)	*Ubiquitin carboxyl-terminal hydrolase MINDY-1.* This hydrolase can remove 'Lys-48'-linked conjugated ubiquitin from proteins. It has exodeubiquitinase activity and has a preference for long polyubiquitin chains, and it may then be a regulator of protein turnover.	Proteostasis
FSD1 (8)	*Fibronectin type III and SPRY domain-containing protein 1.* The protein is probably involved in microtubule organization and stabilization.	Microtubule
LRRC1 (8)	*Leucine-rich repeat-containing protein 1.* This is a cytosolic protein.	Cytosol
SENP1 (8)	*Sentrin-specific protease 1.* The encoded cysteine protease specifically targets members of the small ubiquitin-like modifier (SUMO) protein family. This protease regulates SUMO pathways by deconjugating sumoylated proteins. This protease also functions to process the precursor SUMO proteins into their mature form.	Sumoylation
CEP55 (8)	*Centrosomal protein of 55.* Intracellular protein involved in cell cycle regulation.	Cell cycle regulation
ODAPH (8)	*Odontogenesis associated phosphoprotein.* The gene encodes an extracellular matrix acidic phosphoprotein.	Extracellular matrix
ZNF22 (8)	*Zinc finger protein 22.* This DNA binding protein is involved in transcriptional regulation.	Transcription
TTC26 (9)	*Intraflagellar transport protein 56.* The protein is a component of the intraflagellar transport (IFT) complex B required for transport of proteins; the protein is involved in microtubule organization.	Intracellular transport<br>Microtubule
TXNDC11 (9)	*Thioredoxin domain-containing protein 11.* The protein possibly acts as a redox regulator involved in DUOX proteins folding.	Proteostasis?
HSP90AB3P (9)	*Putative heat shock protein HSP 90-beta-3.*	
RNLS (9)	*Renalase.* The protein catalyzes the oxidation of the less abundant 1,2-dihydro-beta-NAD(P) and 1,6-dihydro-beta-NAD(P) to form beta-NAD(P)(+). The enzyme hormone is secreted	Oxidoreductase.

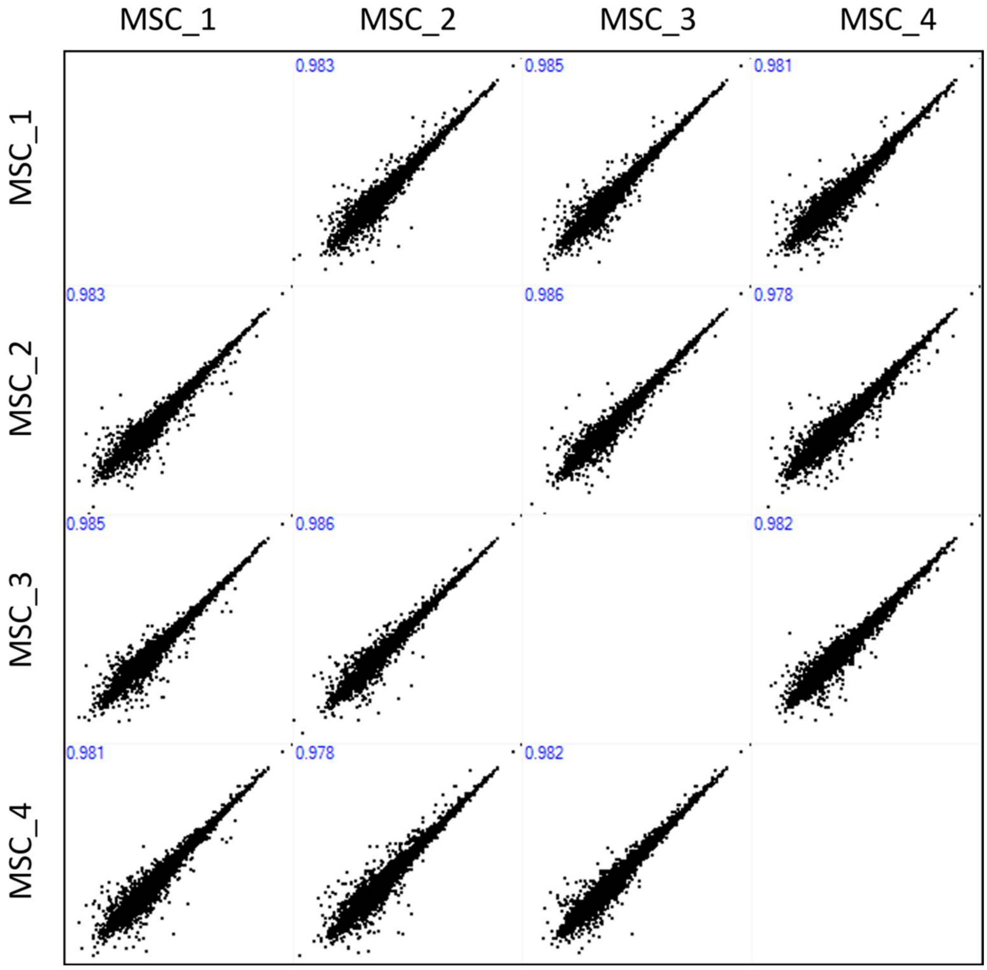

Figure S1. Correlation of the proteomic MSC profile; a detailed presentation of the results for the four MSC medium controls, i.e. MSC cultures prepared in medium alone in four independent cultures. The Pearson R correlation coefficient is indicated for each correlation analysis.

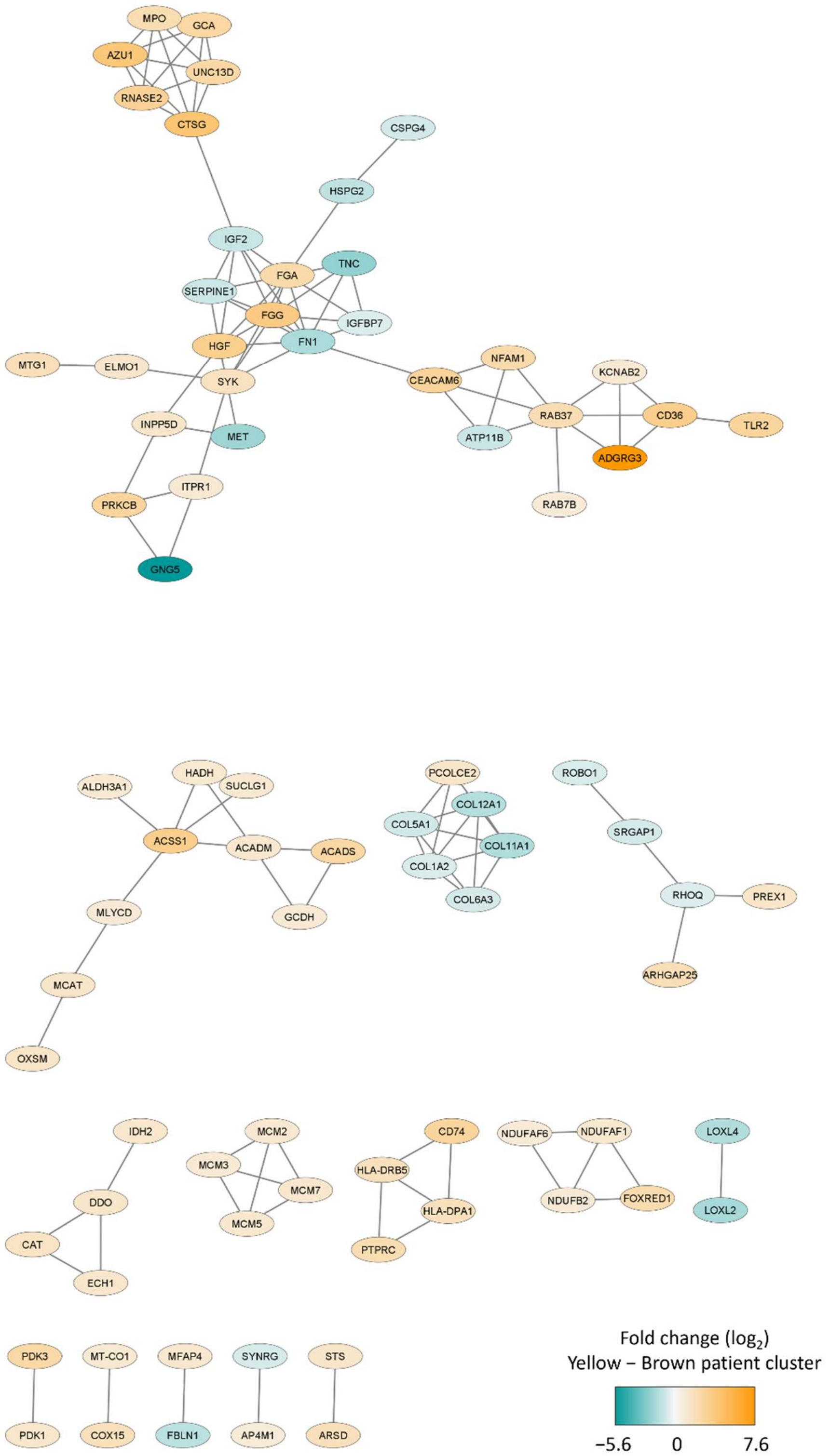

Figure S2. A protein-protein interaction network analysis based on the 217 MSC proteins that differed significantly between the two main patients subset in the unsupervised hierarchical cluster analysis presented in Figure 2. The original network was generated in the String database and imported to Cytoscape for processing and color coding. The color coding of the protein nodes indicates the log_2-transformed protein fold change, where green indicates increased protein abundance and brown indicates decreased protein abundance (i.e. increased in the brown main patient color) in the upper yellow main patient cluster (n=10) identified in Figure 2 in the article.

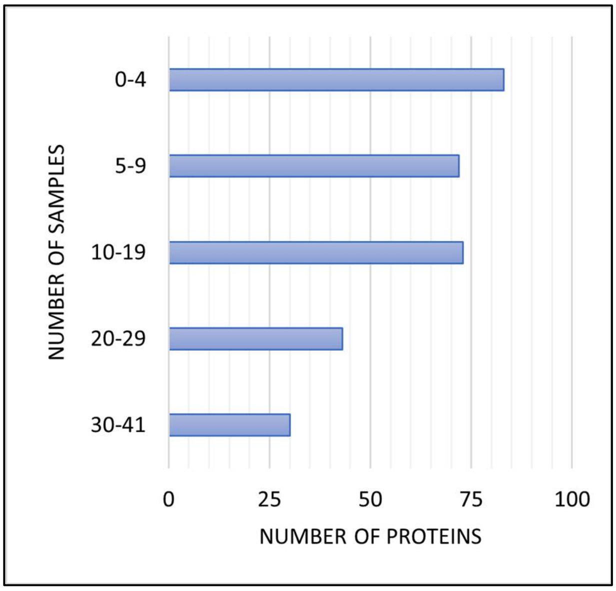

Figure S3. Proteins only quantified when MSCs were cultured in the presence of AML conditioned medium; 301 proteins were identified. The figure shows the number of samples/ patients (y-axis, number of samples) and the corresponding number of the 301 proteins (x-axis) that could be quantified for the indicated sample/patient number on the y-axis.

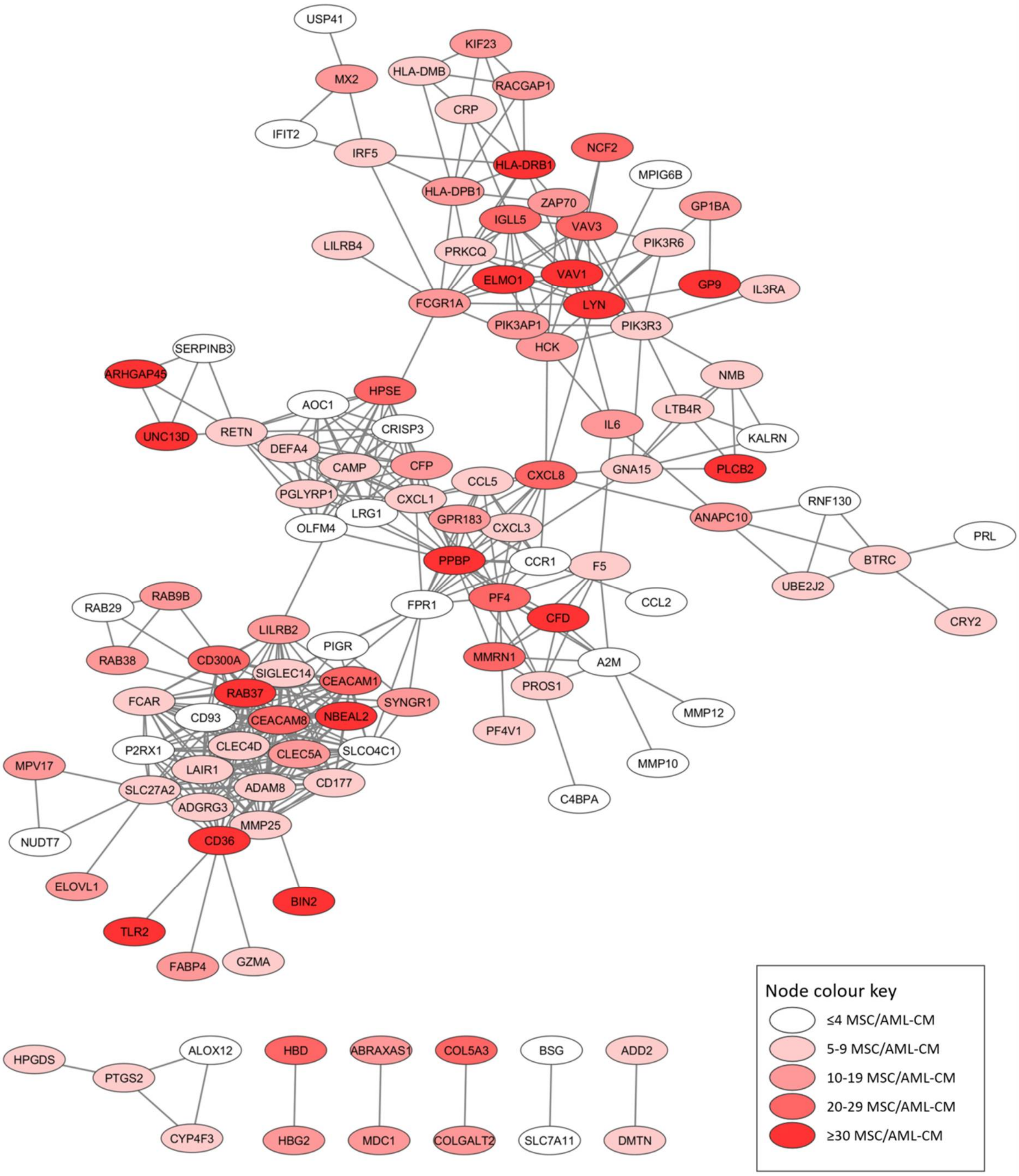

Figure S4. A protein-protein interaction network analysis based on the 301 proteins that were only quantified for MSCs cultured with AML conditioned medium but not for any of the control MSCs cultured in medium alone. The original network was generated in the String database and imported to Cytoscape for processing and color coding. The number of patients/samples (i.e. MSC samples incubated with AML conditioned medium, AML-CM) with quantified levels for each individual protein is indicated to the lower right in the figure.

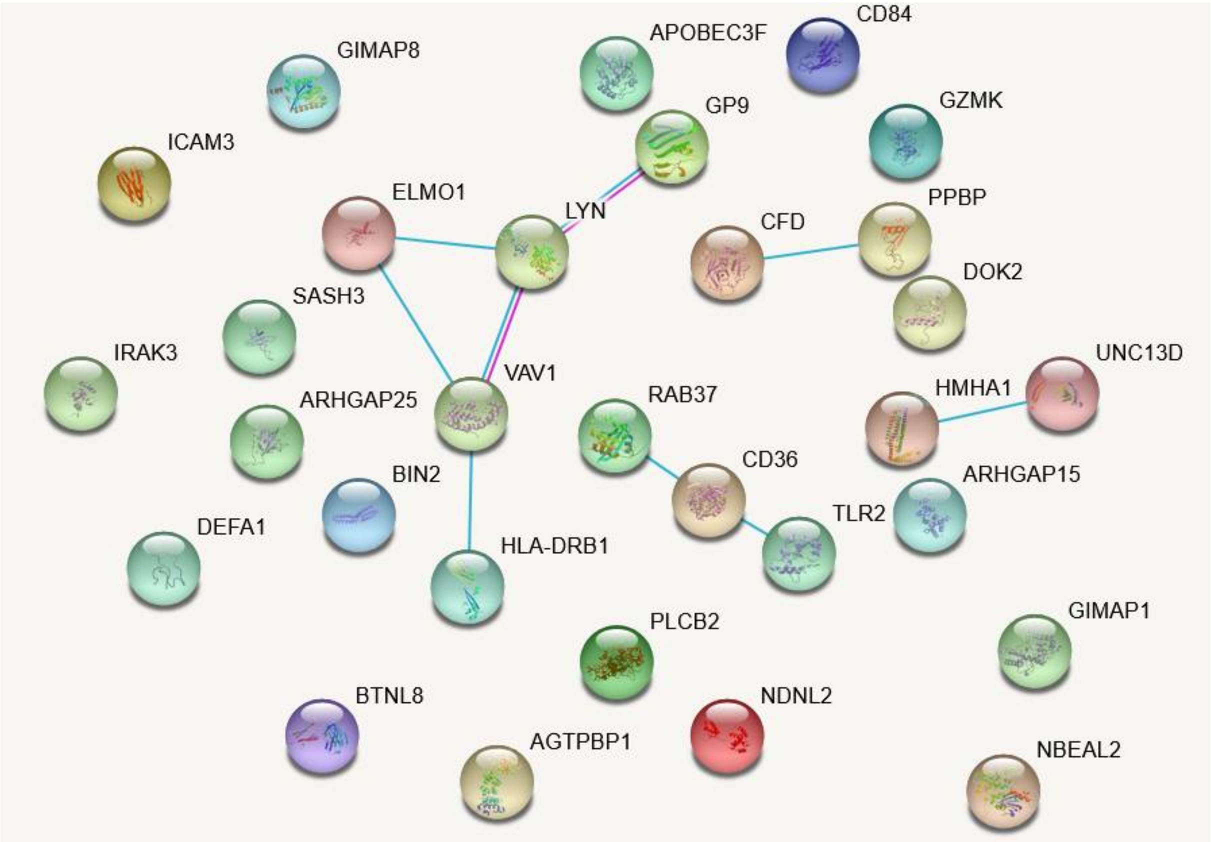

Figure S5. A protein-protein interaction network analysis based on 30 proteins that were quantified for at least 30 MSC samples derived from cultures supplemented with AML conditioned medium, but not for any of the four control MSCs cultured in medium alone.

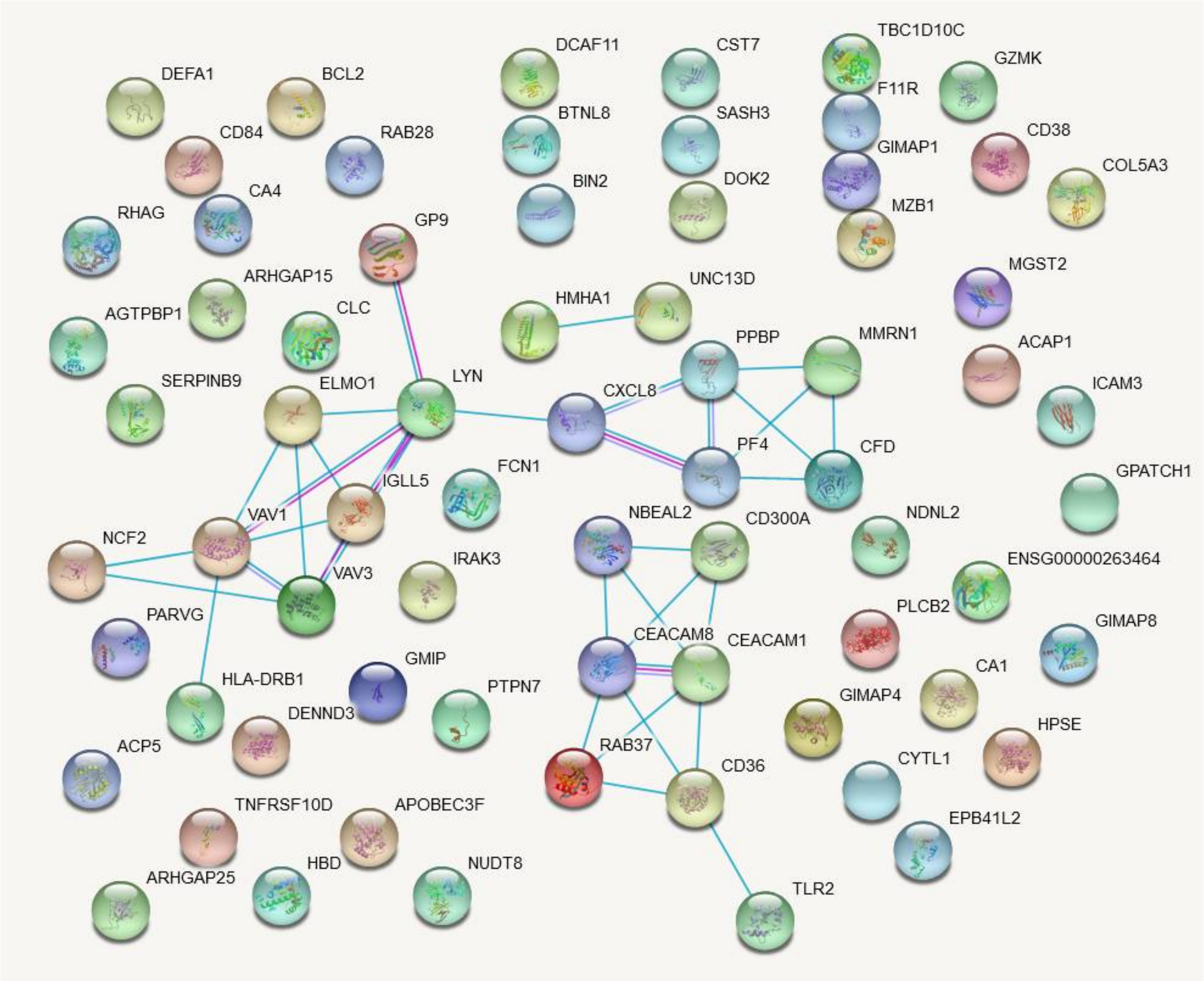

Figure S6. A protein-protein interaction network analysis based on all proteins that were quantified for at least 20 MSC samples derived from cultures supplemented with AML conditioned medium, but not for any of the four control MSCs cultured in medium alone.

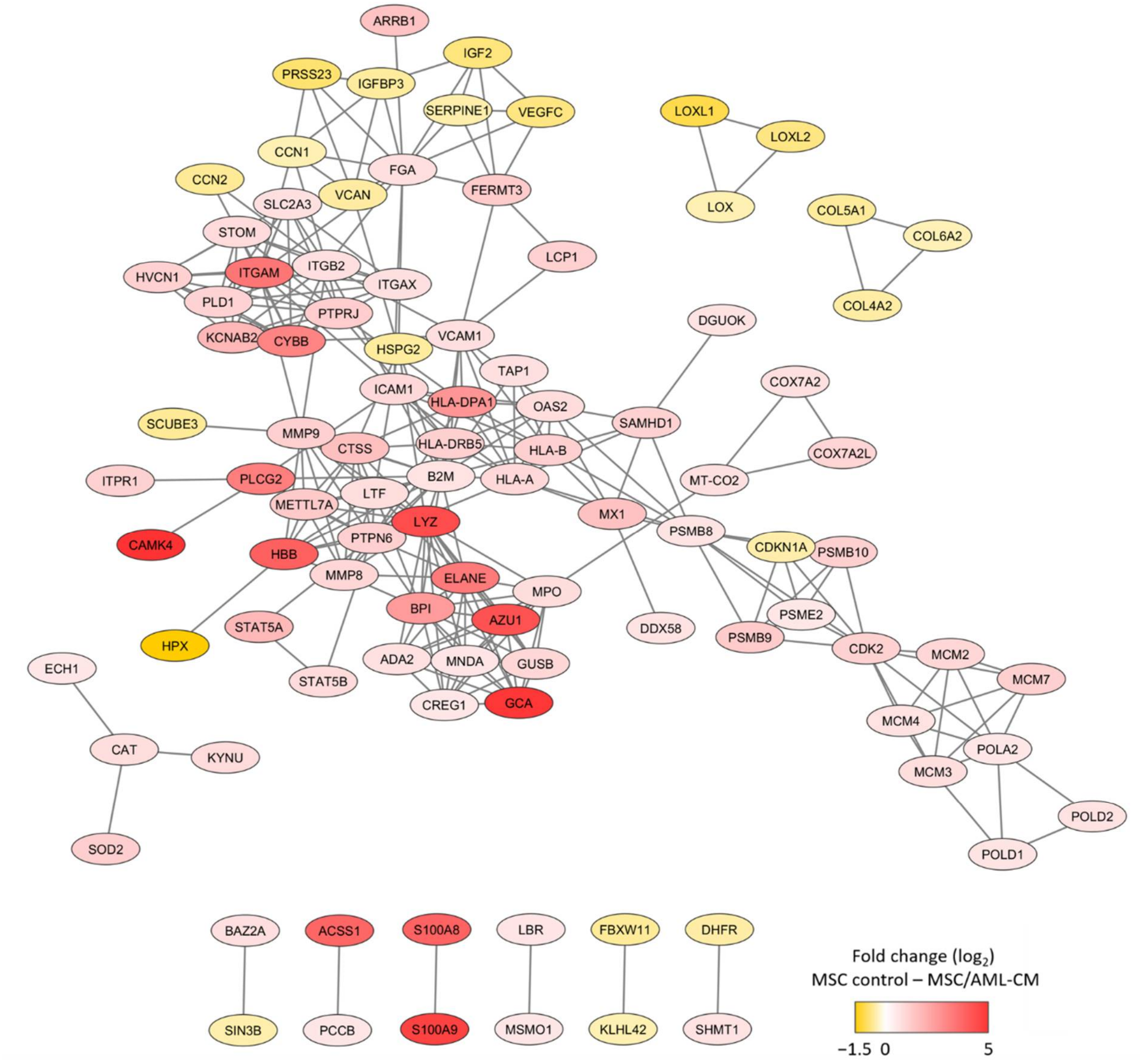

Figure S7. A protein-protein interaction network analysis based on the 201 MSC proteins that were significantly altered for MSCs cultured with AML conditioned medium (AML-CM). The original network was generated in the String database and imported to Cytoscape for processing and color coding. The color coding of the protein nodes indicates the log_2-transformed protein fold change, where yellow indicates decreased protein abundance and red indicates increased protein abundance in MSCs after exposure to AML conditioned medium relative to MSC medium controls.
